# Comparison of the indirect immunofluorescence assay and a commercial ELISA to detect KSHV antibodies

**DOI:** 10.1128/spectrum.01186-24

**Published:** 2024-09-23

**Authors:** Valentin Leducq, Léa Ben Said, Sophie Sayon, Vincent Calvez, Anne-Geneviève Marcelin, Aude Jary

**Affiliations:** 1 Sorbonne Université, INSERM, Institut Pierre Louis d'Epidémiologie et de Santé Publique (iPLESP), AP-HP, Hôpital Pitié-Salpêtrière, Service de Virologie, Paris, France; University of Wisconsin-Madison, Madison, Wisconsin, USA

**Keywords:** KSHV, HHV-8, IFA, ELISA, method comparison

## Abstract

Kaposi’s sarcoma-associated herpesvirus (KSHV) is an oncogenic virus involved in several diseases. The gold standard for KSHV sero diagnosis remains the indirect immunofluorescence assay (IFA), which is time-consuming and operator-dependent. We compared this method with an enzyme-linked immunosorbent assay (ELISA) targeting solubilized KSHV whole-genome extract among positive (*n* = 49, including 76% of HIV-infected patients) and negative (*n* = 14) control groups. We also included 14 sera with equivocal IFA results. ELISA showed better performance in detecting KSHV antibodies (McNemar’s test, *P =* 0.0455). The sensitivity and specificity of both methods were 79% (64–89) and 100% (66–100) for the IFA, respectively, and 94% (83–99) and 100% (66–100) for ELISA, respectively. All IFA equivocal results were either negative or positive with ELISA. ELISA is more reliable and could be a good alternative for determining KSHV serological status, particularly in the context of immunocompromised patients and equivocal serology with the IFA.

**IMPORTANCE:**

Kaposi’s sarcoma-associated herpesvirus (KSHV) sero status remains challenging because no perfect reference is available for the detection of KSHV antibodies. The current gold-standard method, the indirect immunofluorescence assay (IFA), has a very good specificity of close to 100%, but a lower sensitivity of around 80–85%, which decreases to 64–67% in immunocompromised patients. Additionally, this method is time-consuming and operator-dependent compared with new serological assays such as the enzyme-linked immunosorbent assay (ELISA). Thus, further research is still needed to improve KSHV sero diagnosis. Here, we compare the KSHV IgG ELISA kit assay (Advanced Biotechnologies Inc) with the gold-standard IFA, targeting the LANA-1 protein from latent BC-3 cell lines.

## OBSERVATION

Kaposi sarcoma-associated herpesvirus (KSHV) is an oncogenic virus involved in Kaposi’s sarcoma (KS), multicentric Castleman disease, and primary effusion lymphoma (PEL). More than 50% of the population is infected with KSHV in the sub-Saharan African region, between 10% and 20% in Eastern Europe and the Mediterranean basin, and less than 5% in Western Europe and North Africa (1). Serological evaluation of KSHV infection relies on various methods to detect KSHV antibodies such as (i) the immunofluorescence assay (IFA), which remains the gold standard, using either latent or lytic PEL cell lines (2, 3); (ii) the enzyme-linked immunosorbent assay (ELISA) with a reactivity against one latent and/or one lytic antigens, the most commonly used being the LANA-1 (ORF73) and the K8.1 proteins, respectively (3); and (iii) more recently, by a multiplex bead-based assay with six KSHV antigens, including ORF73, K8.1, ORF38, ORF59, ORF61, and ORF65 (4). Some issues still remain with the gold-standard method, which shows low sensitivity, especially in HIV-infected patients, when working with latent PEL cell lines, or higher sensitivity but lower specificity with previously stimulated PEL cell lines, due to cross-reactivity with other antigens (5). For instance, in AIDS patients with KS, the sensitivity of IFA against latent antigen ranges from 64% to 67% (5, 6).

For this study, 77 sera were tested, including 49 samples from patients with KSHV diseases (positive control group and negative control group) and 14 samples from patients negative for IgG antibodies with the IFA (negative control group). We also included 14 samples from patients reported as equivocal with the IFA. First, sera were tested for the detection of IgG antibodies against LANA-1, with the IFA using slides coated with unstimulated BC-3 cells line chronically infected with KSHV (2). This method has a sensitivity of around 80%–85% but a very high specificity, close to 100%. Results were reported as positive, negative, or equivocal when fluorescence was ambiguous. Then, sera were tested with a KSHV IgG ELISA (Advanced Biotechnologies Inc), which is intended for the detection of KSHV IgG antibodies using solubilized KSHV whole-genome extract. The experiment was performed according to the manufacturer’s procedures, and the color intensity was proportional to the KSHV IgG level. The results were referred to as negative when optical density (OD) ratio ≤0.75, positive when OD ratio ≥1.00, and equivocal when 0.75 < OD ratio < 1.00. We assessed the test performance by determining the sensitivity (Se), specificity (Sp), positive predictive value (VPP), and negative predictive value (VPN). McNemar’s chi-square test and Cohen’s kappa coefficient were used to determine whether there was marginal homogeneity between the proportions of positive and negative samples observed using both methods and the degree of agreement between the two methods, respectively.

Sixty-three sera from the negative and positive control groups were used to compare the methods. The participants’ median (interquartile [IQR]) age was 53 (39-64.5) years, most of them being male (79%) (Table 1). Thirty-six had KS, including 24 epidemic KS and 10 iatrogenic KS (two for which the KS epidemiological form was unknown). Thirteen patients had hematological diseases, including 11 MCD and 2 PEL, of whom 11/13 were also diagnosed with KS. Among this population, 76% were HIV-infected. Fourteen patients tested negative for KSHV antibodies with IFA, of whom nine were free from KSHV diseases. For all patients, KSHV sero diagnosis was performed in the context of post-solid organ transplantation (SOT) follow-up, and they were HIV-negative. For the five remaining patients, information about KSHV diseases was unknown, as well as HIV status.

**TABLE 1:** Characteristics of the studied population^*c*^

	All participants (*n* = 63)	Positive control group (*n* = 49)	Negative control group (*n* = 14)
Age, median (IQR)	53 (39–64.5)	38 (26–62)	51 (33–68)
Male, *n* (%)	50 (79)	44 (90)	6 (43)
HIV-positive, *n* (%)^*a*^	35 (76)	35 (76)	0 (0)
KSHV-associated diseases^*b*^	49 (78)	49 (100)	0 (0)
Kaposi’s sarcoma	47 (75)	47 (96)	0 (0)
Multicentric Castleman disease	11 (17)	11 (22)	0 (0)
Primary effusion lymphoma	2 (3)	2 (4)	0 (0)

^*a*^ Three patients in the positive control group and five patients in the negative control group whose HIV status is unknown.

^*b*^ Nine patients in the negative control group with clinical information available.

^*c*^ IQR, interquartile range.

**FIG 1:**
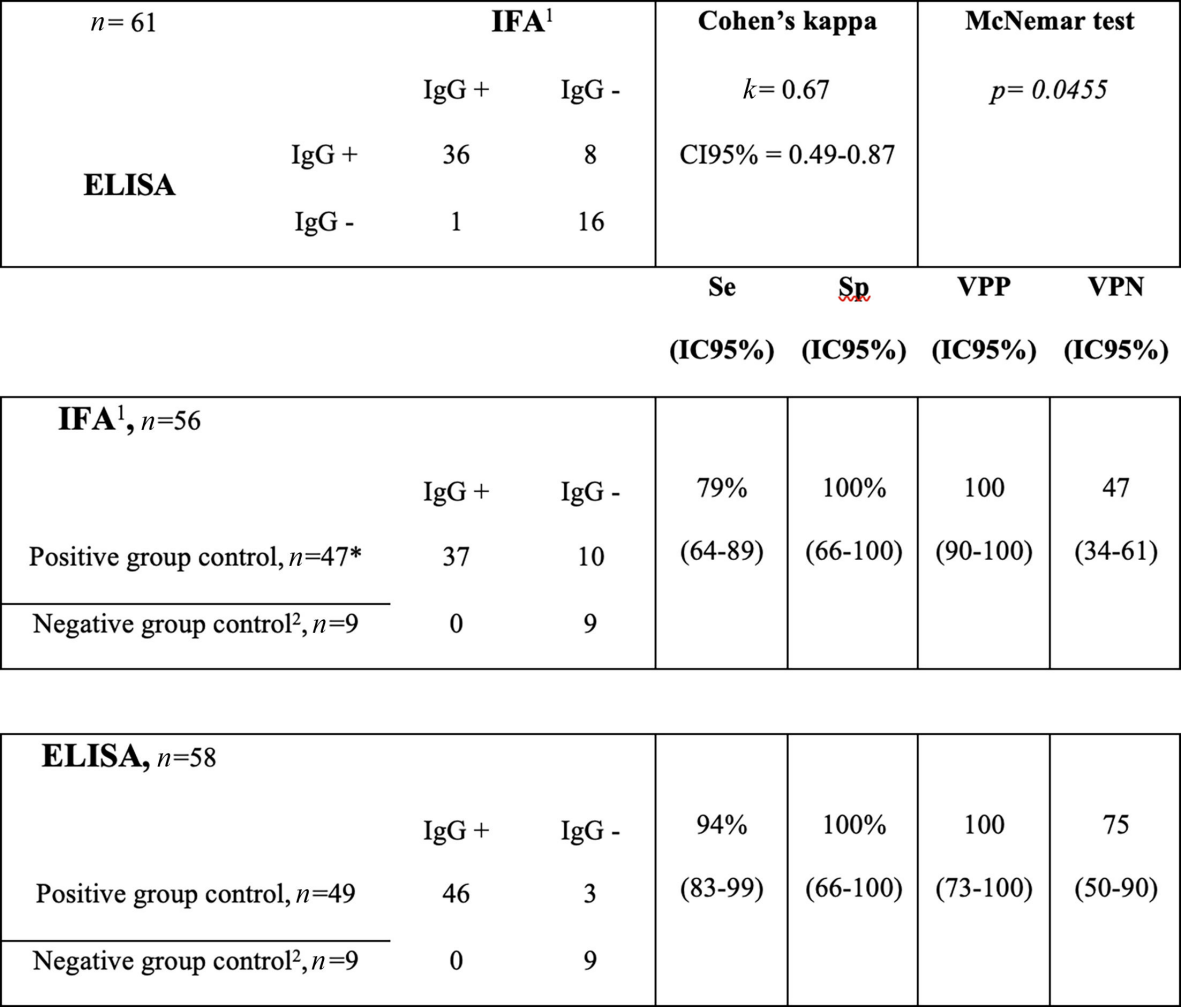
Performance and concordance of the two assays to detect KSHV antibodies. ^1^Two samples referred to as equivocal with the IFA were excluded from the analysis. ^2^We excluded five samples from the negative control group for which clinical information about KSHV diseases was not available. IgG+, immunoglobulin G positive for KSHV antibodies; IgG–, immunoglobulin G negative for KSHV antibodies.

In the positive control group, positive KSHV antibodies were detected in 37 (76%) cases with the IFA and in 46 (94%) cases with ELISA (*P =* 0.038). Eight (16%) samples were negative with the IFA and positive with ELISA (four HIV-positive and four SOT patients), while only one sample (2%) was positive with the IFA and negative with ELISA (Fig. 1). Two samples were negative with both methods, and two sera were equivocal with the IFA but positive with ELISA. Among positive sera, the median of OD ratio obtained with ELISA was 8.7 (3.5–8.9). According to the HIV status, the proportion of positive sera was different with the IFA (88% in HIV-positive patients versus 45% in HIV-negative patients, *P =* 0.008) but not with ELISA (94% in HIV-positive patients versus 91% in HIV-negative patients, *P >* 0.99). Within the negative control group, all sera were also negative with ELISA. Among these, the median of OD ratio was 0.09 (–0.03–0.15) and was significantly lower compared with KSHV-positive sera (*P <* 0.0001). None of them were equivocal.

The performance to detect KSHV antibodies was significantly different between the two methods (McNemar’s test, *P =* 0.0455), and there was a substantial agreement between the results obtained (kappa = 0.67, 95% confidence interval [CI] 0.49–0.86). The clinical Se and Sp to detect KSHV antibodies were 79%(64–89) and 100% (66–100) for the IFA, respectively, and 94% (83–99) and 100% (66–100) for ELISA, respectively (Fig. 1).

All the equivocal samples with the IFA were reported as either negative (9/14) or positive (5/14) by ELISA. The OD ratio was high for all the KSHV-positive antibody measurements, ranging from 6.12 to 8.84, except for one serum with a weak OD ratio of 1.34. The remaining sera were considered negative with an OD ratio ranging from −0.33 to 0.18. Clinical characteristics were not available for any of them.

Here, we compared the gold-standard IFA and ELISA to determine KSHV serology. We found that ELISA had a better sensitivity and a similar specificity than the IFA. Interestingly, ELISA was less impacted by the immunosuppression than the IFA.

The sensitivity (79%) and specificity (100%) of the IFA were found to be similar to a previous report in the literature (3), with an underdiagnosis of KSHV antibodies in HIV-infected and SOT patients. Since these two particular populations have the highest risk of developing KSHV-associated diseases, that is, the incidence of KS is 200 times higher in SOT patients compared with the general population (7), it is important to use a better serological assay in terms of performance but also in terms of technical execution. Indeed, the IFA is a manual test that relies on the operator performing the technique and is a time-consuming procedure. Compared with the IFA, ELISA was significantly better at detecting KSHV antibodies (94% versus 79% in the positive control group), with a specificity of 100%. This assay could be partially automatized and has the advantage of being independent of microscopic slide reading, making it more reliable (8). Previous work on in-house and commercial ELISA targeting the whole virus also reported an increase in sensitivity (87% and 82%, respectively) compared with in-house IFA with similar or lower specificity (100% and 88%, respectively) (3). In this report, they performed latent class analysis because no perfect reference is currently available for KSHV antibodies. Interestingly, the 14 sera with equivocal results with the IFA were found to be either positive or negative with ELISA. Regarding diagnosis, from our experience, equivocal IFA results are not rare, especially in patients with pre-control or follow-up of SOT, probably due to medication. ELISA could therefore be a good alternative in that context.

Our study had some limitations: (i) we only included a small number of patients, especially in the negative control group, for whom clinical information was available for only half of them, and (ii) we cannot rule out that some of the participants in the negative control group were immunocompromised and therefore negative for KSHV antibodies because of immunosuppression.

In conclusion, ELISA could be a good alternative for determining the KSHV serological status, particularly in cases of equivocal serology with the IFA or among immunocompromised patients.

## Data Availability

The data that support the findings of this study are available upon reasonable request.
